# Bioprospecting for industrially relevant exopolysaccharide-producing cyanobacteria under Portuguese simulated climate

**DOI:** 10.1038/s41598-023-40542-6

**Published:** 2023-08-21

**Authors:** José Diogo Cruz, Cédric Delattre, Aldo Barreiro Felpeto, Hugo Pereira, Guillaume Pierre, João Morais, Emmanuel Petit, Joana Silva, Joana Azevedo, Redouan Elboutachfaiti, Inês B. Maia, Pascal Dubessay, Philippe Michaud, Vitor Vasconcelos

**Affiliations:** 1 Faculty of Sciences, University of Porto, Rua do Campo Alegre, 4169-007 Porto, Portugal; 2 Interdisciplinary Center of Marine and Environmental Research (CIIMAR/CIMAR), University of Porto, Terminal de Cruzeiros do Porto de Leixões, Avenida General Norton de Matos, S/N, 4450-208 Matosinhos, Portugal; 3 Université Clermont Auvergne, Clermont Auvergne INP, CNRS, Institut Pascal, 63000 Clermont-Ferrand, France; 4 Institut Universitaire de France (IUF), 75005 Paris, France; 5 GreenCoLab - Associação Oceano Verde, Universidade do Algarve, Campus de Gambelas, 8005-139 Faro, Portugal; 6 UMRT INRAE 1158 BioEcoAgro, BIOlogie des Plantes et Innovation (BIOPI), Université de Picardie Jules Verne, IUT d’Amiens, Avenue des Facultés, Le Bailly, 80025 Amiens, France; 7 R&D Department, Allmicroalgae Natural Products S.A, Rua 25 de Abril 19, 2445-287 Pataias, Portugal; 8 CCMAR – Centre of Marine Sciences, University of Algarve, 8005-139 Gambelas, Faro, Portugal

**Keywords:** Biochemistry, Biotechnology, Microbiology

## Abstract

Cyanobacterial exopolysaccharides (EPS) are potential candidates for the production of sustainable biopolymers. Although the bioactive and physicochemical properties of cyanobacterial-based EPS are attractive, their commercial exploitation is limited by the high production costs. Bioprospecting and characterizing novel EPS-producing strains for industrially relevant conditions is key to facilitate their implementation in various biotechnological applications and fields. In the present work, we selected twenty-five Portuguese cyanobacterial strains from a diverse taxonomic range (including some genera studied for the first time) to be grown in diel light and temperature, simulating the Portuguese climate conditions, and evaluated their growth performance and proximal composition of macronutrients. *Synechocystis* and *Cyanobium* genera, from marine and freshwater origin, were highlighted as fast-growing (0.1–0.2 g L^−1^ day^−1^) with distinct biomass composition. *Synechocystis* sp. LEGE 07367 and Chroococcales cyanobacterium LEGE 19970, showed a production of 0.3 and 0.4 g L^−1^ of released polysaccharides (RPS). These were found to be glucan-based polymers with high molecular weight and a low number of monosaccharides than usually reported for cyanobacterial EPS. In addition, the absence of known cyanotoxins in these two RPS producers was also confirmed. This work provides the initial steps for the development of cyanobacterial EPS bioprocesses under the Portuguese climate.

## Introduction

Cyanobacteria and microalgae are major contributors to the bio-economy by sourcing feedstocks for food, feed, bioenergy, cosmetics and bioplastics^1^. Additionally, significant research & development efforts are leveraging the current industrial production of these photoautotrophic factories into economically feasible applications^2^. Certain products, such as biopolymers (i.e. polymeric susbtances of natural origin), are still limited by the production cost and capacity when considered for bulk markets. There are, however, high added value markets, for particular applications where cyanobacteria/microalgae biopolymers can be successfully commercialized due to their specific physicochemical and/or bioactive properties^3–5^. The relevance of these biopolymers in the bio-economy can be hailed through technological improvements and the discovery of new photoautotrophic factories^1^. Thus, bioprospecting for industrially relevant conditions (e.g. light, temperature) is key for bioprocess development and cost reduction^6,7^.

Cyanobacteria are a promising source of biopolymers in the form of polysaccharides, which are found intracellularly (e.g. glycogen) or extracellularly as exopolysaccharides (EPS)^8–10^. EPS are polysaccharide-rich exudates that stick to the cell wall's outer side. However, they also exist in the supernatant, not connected to the cell wall. Although the origin of these polysaccharides in the supernatant is not fully understood, evidence suggests that they have similar monosaccharides composition^11–14^. However, these two phases are extracted differently and are distinguished as cell-bound polysaccharides (CPS) and released polysaccharides (RPS)^15^.

These biopolymers have been intensively studied, and some showed interesting techno-functional properties^16–18^; with bioactive properties such as anti-viral, anti-inflammatory and wound healing properties^19–21^ making them good candidates for high added-value products.

A comprehensive review on the methodologies applied to bioprospecting cyanobacterial EPS showed common features: light and temperature profiles are set constant, and strains explored are predominantly from Nostocales and Oscilatoriales orders and *Nostoc* genus^15^. These studies showed that EPS content is a strain-specific trait.

The biological diversity present in culture collections can be a source of raw material for the exploration of biotechnological applications^22^. The Blue Biotechnology and Ecotoxicology Culture Collection (LEGE-CC) is a biobank of cyanobacteria and microalgae. Currently comprises more than 700 non-axenic cyanobacterial strains, 80% isolated from Portuguese environments^23^.

Bioprospecting under process-oriented conditions is a useful way to determine critical process factors and use them to discover adapted microbial resources, at the same time, get closer to industrial scenarios^6,24^. Portugal has pioneered microalgae production and commercialization in Europe, having the oldest production unit, and now this segment is well represented by several industrial and artisanal producers^25^. The Portuguese climate has compatible conditions with the ones necessary for the industrial production of cyanobacteria.

In this context, non-axenic cyanobacterial strains have been screened for relevant industrial conditions to determine and characterize their potential as a source of EPS biopolymers and non-toxic biomass for biotechnological applications. For that, strains were initially evaluated regarding their productivity and protein content when grown in photobioreactors (PBR) simulating the central Portugal spring diel light and temperatures. Then, the biomass of most productive strains under these criteria were profiled for their proximate composition (protein, carbohydrates, lipids and ashes). Strains producing RPS were prioritized. Those were checked for the presence of cyanotoxins through the detection of genes involved in the biosynthesis of these known toxins through traditional molecular methods and confirmed through ESI–LC–MS/MS. Their polysaccharides were partially characterized through GC/MS-EI of trimethylsilyl-*O-glycosides* for monosaccharides composition and HPSEC-MALS for molecular weight estimation.

## Results

### LEGE-CC holds EPS producing strains

A pre-screening of sixty-three cyanobacterial strains (data not shown) allowed the selection of twenty-five strains through the staining of neutral polysaccharides using Alcian blue formulation. A qualitative characterization of these twenty-five strains is presented in Supplementary Material (SM) Table S1. Overall, neutral polysaccharides were mostly attached to the cellular structure, as CPS, while some had no closeness to the cyanobacterial cells, belonging to the RPS fraction.

**Figure 1:**
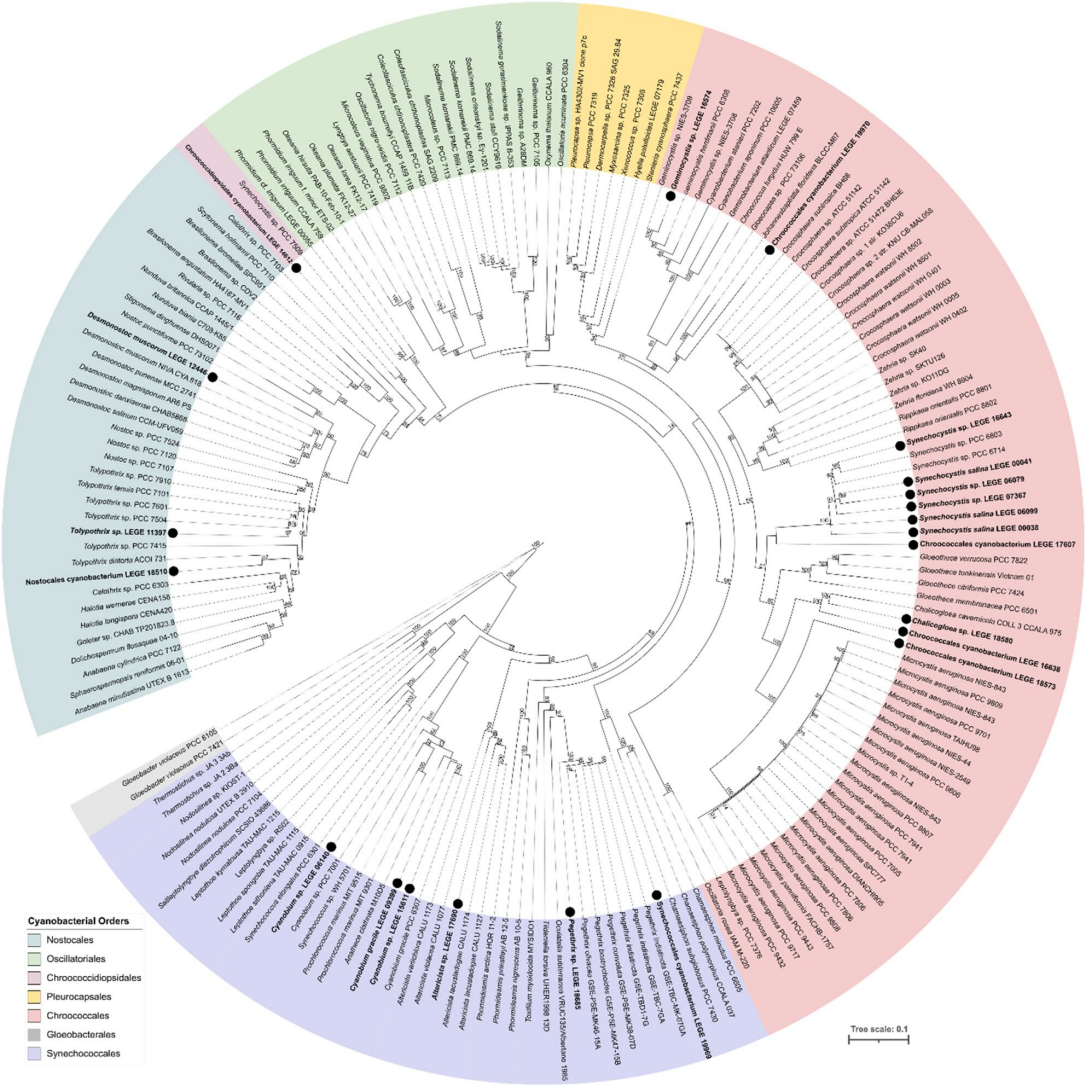
Maximum likelihood phylogenetic tree based on 183 partial 16S rRNA gene sequences of cyanobacteria. *Gloeobacter violaceus* PCC 7421 and *G. violaceus* PCC 8105 were used as the outgroup. LEGE-CC strains used in this work are indicated in bold and with a black circle. The different colour segments represent strain placement at the order level following^26^. Bootstrap values over 50% are indicated at the nodes.

The identification of the selected strains (SM Table S2) shows high diversity among them, comprising five orders: Chroococcales (54%), Synechococcales (25%), Nostocales (13%), Chroococcidiopsidales (4%) and Oscillatoriales (4%). The study sample, shown in Fig. 1, comprises cyanobacteria from diverse habitats: freshwater environment (81%), marine origin (15%) and terrestrial (4%).

## Strain productivity under simulated Portuguese climate

The selected strains of cyanobacteria were exposed to spring-simulated conditions, with a temperature range of 11–22 °C, a maximum light intensity of ~ 1200 µmol m^−2^ s^−1^, and a day length averaged to 14 h. Twenty-one of the twenty-five strains were able to thrive on these culture conditions while four were excluded for not being capable of duplicating their biomass during the fifteenth-day cultivation, namely Chrocodiopsales cyanobacterium LEGE 14612, Chroococcales cyanobacterium LEGE 16638, *Microcystis aeruginosa* LEGE 91353, Synechococcales cyanobacterium LEGE 17617. In addition, other five strains were excluded from the screening due to contamination by microflagelates around day eight of cultivation: *Cyanobium* gracile LEGE 09399, *Geminocystis* sp. LEGE 16574, Chroococcales cyanobacterium LEGE 18573, *Pegethrix* sp. LEGE 18685 and *Synechocystis* sp. LEGE 16643. In sum, nine of the twenty-five strains were excluded from the following steps.

**Figure 2:**
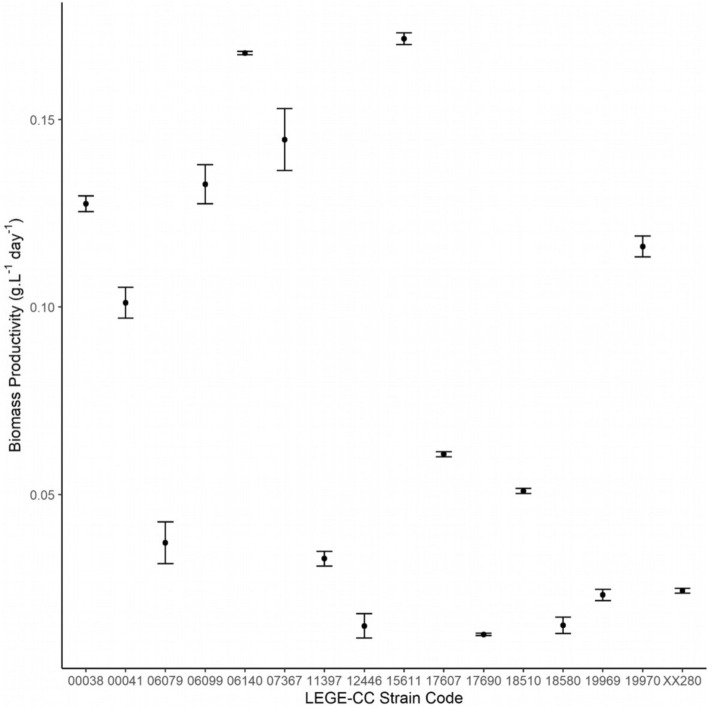
Biomass productivity of cyanobacterial strains for the period of 15 days. Biomass productivity is expressed in g L^−1^ day^−1^ ± standard error (n = 3).

The combination of diel light and temperature directly affected the biomass productivity of cyanobacteria, as shown in Fig. 2. Two virtual groups can be distinguished: a less productive group (< 0.10 g L^−1^ day^−1^) composed of nine strains and a more productive group (≥ 0.1 g L^−1^ day^−1^) consisting of seven strains. Interestingly, the most productive group is composed of all marine strains understudy, mostly belonging to *Synechocystis* and *Cyanobium* genera (*Synechocystis salina* LEGE 000038, *Synechocystis salina* LEGE 00041, *Synechocystis salina* LEGE 06099, *Cyanobium* sp. LEGE 06140) and three freshwater strains (*Synechocystis* sp. LEGE 07367, *Cyanobium* sp. LEGE 15611, Chroococcales cyanobacterium LEGE 19970). Among these, the highest biomass productivity was reached by the two *Cyanobium* strains (Fig. 2).

The least productive group is represented by nine strains from Chroococcales (4), Synechococcales (2), Nostocales (3) and Oscilatoriales (1) orders, namely *Synechocystis* sp. LEGE 06079, *Tolypothrix* sp. LEGE 11397, *Desmonostoc muscorum* LEGE 12446, *Chroococcales* cyanobacterium LEGE 17607, *Altericista* sp. LEGE 17690, Synechococcales cyanobacterium LEGE 19969, *Planktothrix* sp. LEGE XX280, Nostocales cyanobacterium LEGE 18510 and *Chalicogloea* sp. LEGE 18580.

### Protein content

**Figure 3:**
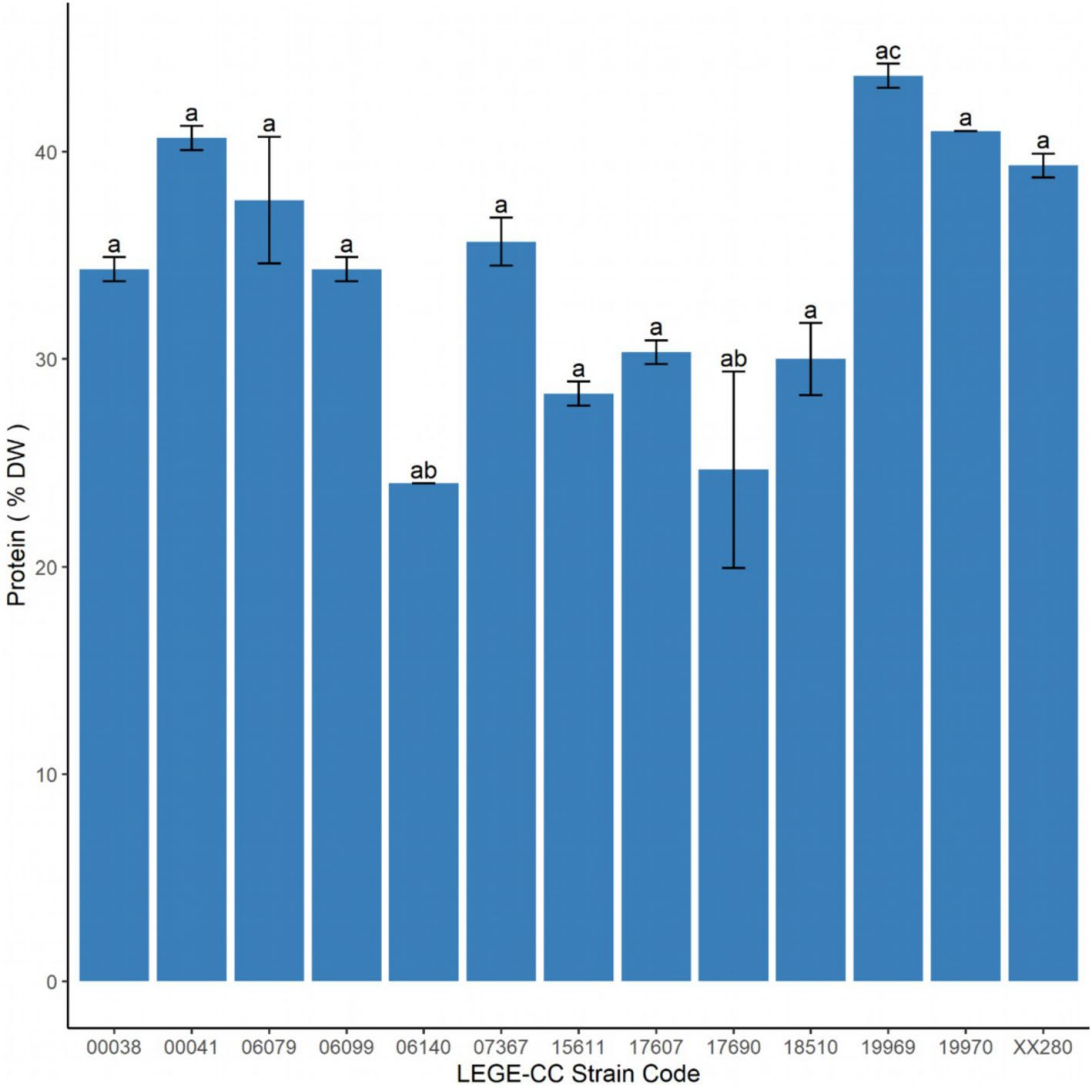
Protein content in percentage of dry weight (DW) of the cyanobacterial strains. Different letters indicate significant differences between strains according to the Nemenyi post-hoc test. Values are given as means ± standard deviation (*n* = *3*).

Figure 3 shows the protein content of cyanobacterial biomass produced in the PBR, except for *Chalicogloea* sp. LEGE 18580, *Tolypothrix* sp. LEGE 11397 and *Desmonostoc muscorum* LEGE 12446, due to insufficient amount of biomass. Overall, the protein content of the cyanobacterial biomass varied from 20 to 40% dry weight (DW), and there were significant differences globally (Kruskal–Wallis test, chi-squared = 36.26; df = 12, *p*-value < 0.001). The post-hoc Nemenyi test found significant differences between the strain LEGE 19969 (the one with higher content, 43% DW) and both LEGE 06140 and LEGE 17690 strains (the two with lower content, 24% and 24.7%, respectively).

The correlation between biomass productivity and protein determined by the Spearman coefficient was negative but non-significant ($\rho$ = − 0.29;* S* = 12,790; *p*-value = 0.07).

## Biomass proximate composition of most productive strains

**Figure 4:**
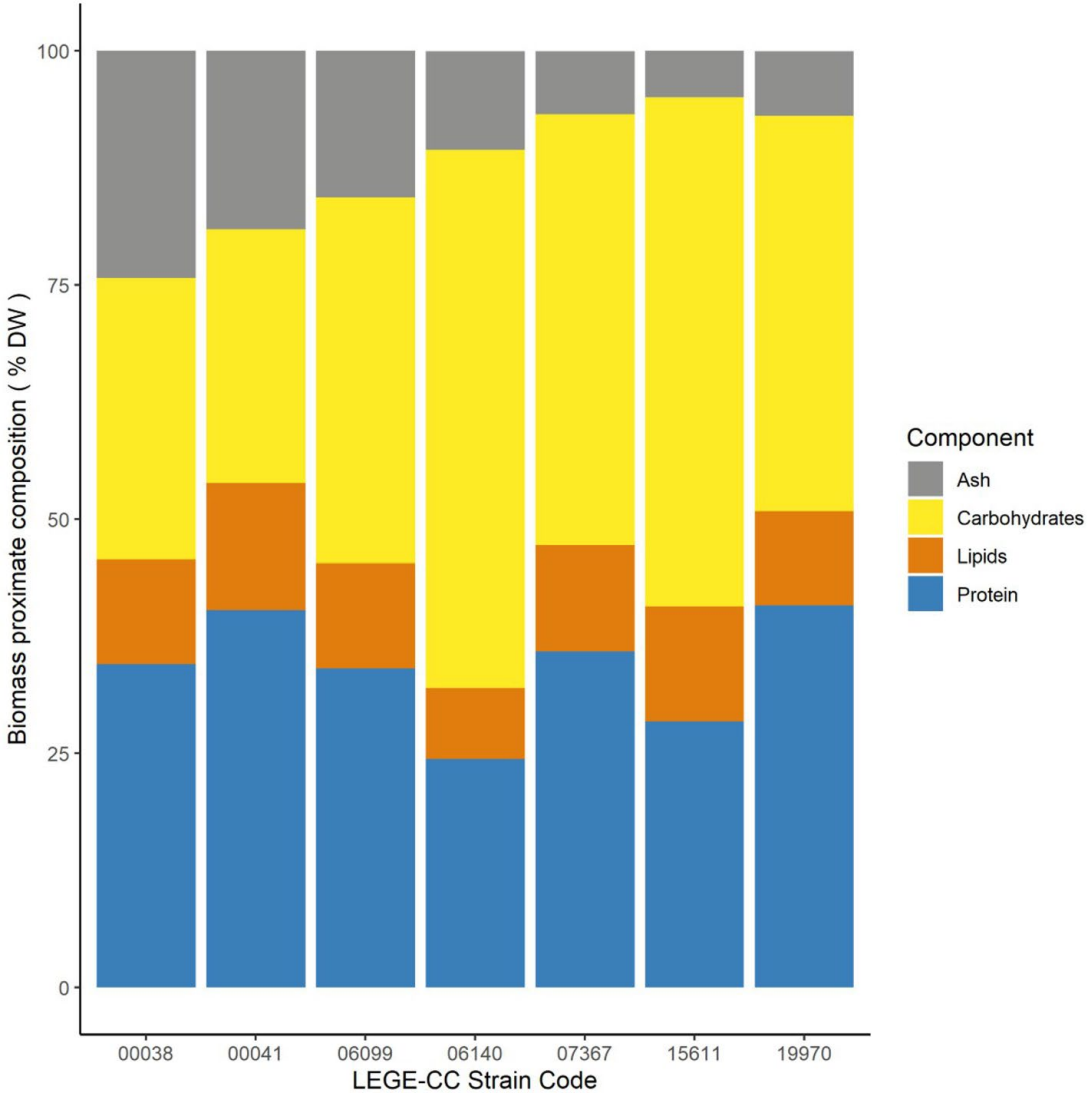
Biomass proximate composition of the most productive cyanobacterial strains (*n* = *3*).

**Figure 5:**
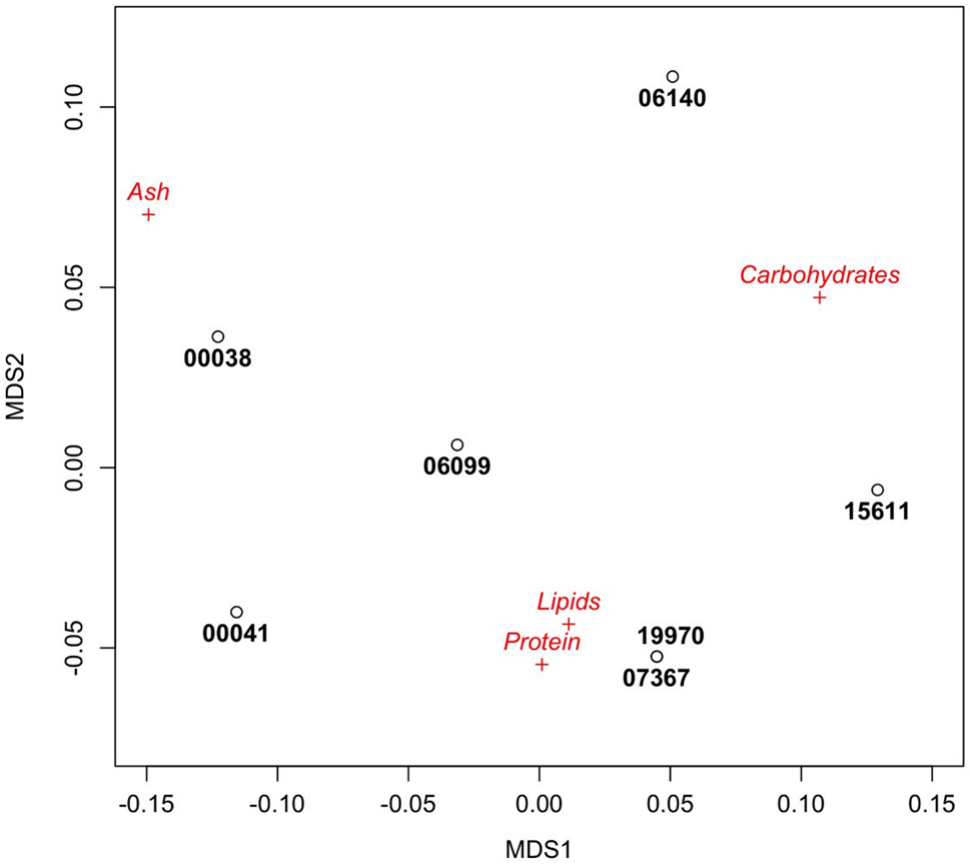
Plot of the non-metric multidimensional scaling for the biochemical composition of the seven strains.

The most productive cyanobacterial strains (four marine and three freshwater) were selected, and a full biomass proximate composition was carried out (Fig. 4). A Non-metric Multidimensional Scaling (NMDS) was applied in other to detect dissimilarity between strains (Fig. 5), presenting a stress value of 9.22 × 10^–5^.

Protein content among these marine and freshwater strains varied from the highest proportions for Chroococcales cyanobacterium LEGE 19970 (41% DW) and the marine *Synechocystis salina* LEGE 00041 (41%), whereas lowest content was observed for marine *Cyanobium* sp. LEGE 06140 (24% DW).

Relative lipid content was lowest for marine *Cyanobium* sp. LEGE 06140 (8% DW) and highest for freshwater *Cyanobium* sp. LEGE 15611 (12% DW) (Fig. 4). *Synechocystis salina* strains (LEGE 00038, LEGE 00041 and LEGE 06099) showed a similar total lipid content (11–12% DW), whereas Chroococcales cyanobacterium LEGE 19970 showed only 10% DW of total lipids.

Ash content for marine strains varied from 10.5% DW (*Cyanobium* sp. LEGE 06140) to 24.3% DW (*Synechocystis salina* LEGE 00038), while among freshwater strains, ash content varied from 5% (*Cyanobium* sp. LEGE 15611) to 6.9% DW (Chroococcales cyanobacterium LEGE 19970).

Carbohydrate content was highest for marine *Cyanobium* sp. (LEGE 06140), representing almost 60% DW, while the lowest value was found for *Synechocystis salina* LEGE 00041 (28.1% DW). Interestingly, freshwater strains averaged higher carbohydrates than marine ones. Additionally, the marine *Cyanobium* sp. LEGE 06140 and freshwater *Cyanobium* sp. LEGE 15611 showed very similar levels of carbohydrate accumulation: 57% DW and 54% DW, respectively. The three *Synechocystis salina* strains (LEGE 00038, 00041 and 06099) showed varying carbohydrate levels (28–39% DW). Chroococcales cyanobacterium LEGE 19970 registered almost even proportions of carbohydrates and protein.

The NMDS analysis (Fig. 5) showed the biomass composition of Chroococcales cyanobacterium LEGE 19970 and *Synechocystis* sp. LEGE 07367 were very similar, with an overlap in their positions (Fig. 5). These two strains are associated with high relative protein and lipid content (Fig. 4). Also, *Synechocystis salina* LEGE 00041 was associated with these two components, but its higher ash content showed a displacement towards this component in the first dimension (Fig. 5). *Synechocystis salina* strain LEGE 00038 was characterized by its relatively high ash content (Fig. 4) and appeared associated to it (Fig. 5), *Synechocystis salina* LEGE 06099 showed the most balanced proportion of all the elements, being the closest to the origin (Fig. 5). *Cyanobium* strains LEGE 15611 and LEGE 06140 were associated with carbohydrates, since they had the largest proportion (Fig. 4).

**Figure 6:**
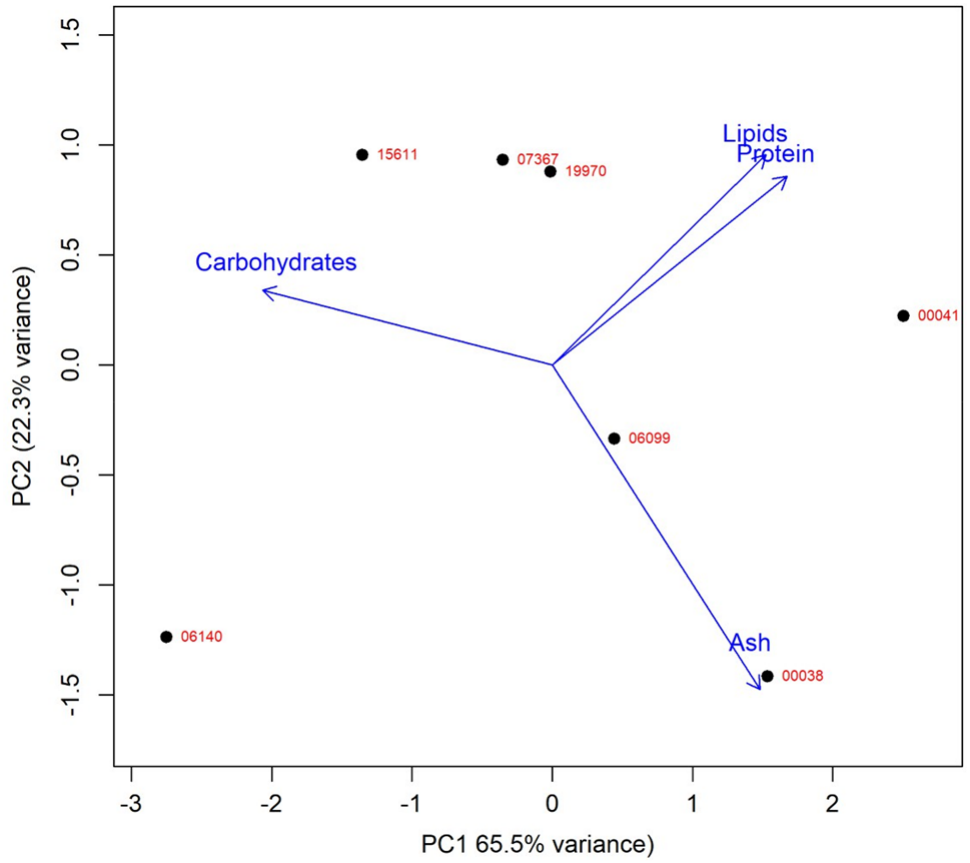
PCA of biochemical components for the most productive cyanobacterial strains.

A Principal Component Analysis (PCA) was built to explore the relationships between the biochemical composition of the most productive strains (Fig. 6). Results of the PC1 explain 65.5% of the total variance. This component was negatively correlated with carbohydrate content and positively with protein, lipid and ash content. The PC2 axis explains 22.3% of the total variance, mostly positively correlated with lipid and protein, and negative with ash. The most remarkable pattern was the negative association between carbohydrate content and all the other components. The PC1 showed the characterization of strains LEGE *Cyanobium* sp. LEGE 15611 and *Cyanobium* sp. LEGE 06140 showed a higher relative carbohydrate content with respect to all other strains; the first was relatively richer in lipids and proteins whereas the second was poorer in lipids and proteins and richer in ash. *Synechocystis salina* LEGE 00041 was more correlated to lipids and proteins. The PC2 confirmed two groups with opposed patterns regarding lipid and protein on one side and ash on the other. *Synechocystis* sp. LEGE 07367 and Chroococcales cyanobacterium LEGE 19970 were richer in proteins and lipids, and *Synechocystis salina* strains (LEGE 00038 and 06099) were richer in ash.

## Partial characterization of RPS

**Figure 7:**
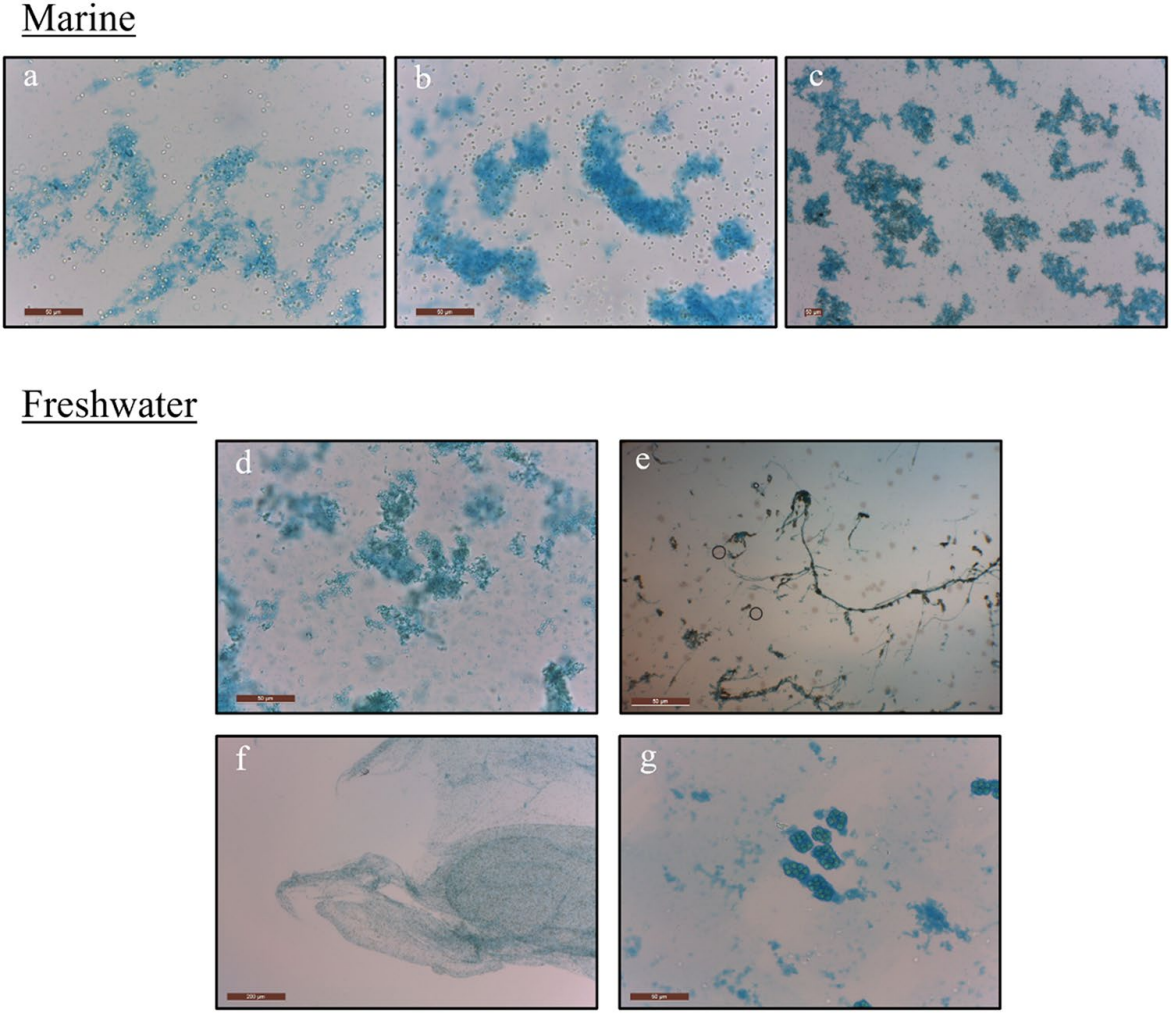
Microphotographs of cyanobacterial EPS after PBR cultivation stained with Alcian Blue formulation clustered by origin. Magnification ranged from 100 to 400× and was adapted to cell size to capture the main profile of EPS around the cells. Letters correspond to: (**a**) *Synechocystis salina* LEGE 00038, (**b**) *Synechocystis salina* LEGE 00041, (**c**) *Synechocystis salina* LEGE 06099, (**d**) *Cyanobium* sp. LEGE 06140, (**e**) *Synechocystis* sp. LEGE 07367 (**f**) *Cyanobium* sp. LEGE 15611 and (**g**) Chroococcales cyanobacterium LEGE 19970.

Neutral polysaccharides at the end of cultivation were stained and microphotographed as shown in Fig. 7. The marine cluster of microphotographs showed a similarity between the EPS staining composed of *Synechocystis salina* strains. Contrarly, the freshwater *Cyanobium* strains showed a difference between the connection of cells and EPS fractions. *Synechocystis* sp. LEGE 07367 showed a layer of mucilage surrounding its cells while the one of Chroococcales cyanobacterium LEGE 19970 showed what we could distinguish between RPS and CPS. EPS production in the form of RPS was only detected for two freshwater strains, *Synechocystis* sp. LEGE 07367 and Chroococcales cyanobacterium LEGE 19970. The volumetric and massic raw RPS yield was higher for the Chroococcales cyanobacterium LEGE 19970, as shown in Table 1.

**Table 1:** Characterization of the volumetric and mass yields of raw RPS obtained from the two freshwater strains.

	*Synechocystis* sp. LEGE 07367	Chroococcales cyanobacterium LEGE 19970
Raw RPS yield (mg L^−1^_supernatant_)	295	432
Raw RPS yield (mg g^−1^_biomass_)	156	295
Raw RPS productivity (mg L^−1^ day^−1^)	20	29
Conductivity (µS cm^−2^)	383	690
Salinity (mg L^−1^) eq. NaCl	183	423
Purification	No	Yes
Mass recovery after purification (%)	100	66.7

### Monosaccharides composition

The compositions in monosaccharides of cyanobacterial RPS from *Synechocystis* sp. LEGE 07367 and Chroococcales cyanobacterium LEGE 19970 were carried out using GC/MS-EI (SM Figure S2). The molar % of monosaccharides detected in the cyanobacterial RPS are shown in Table 2. Both RPS samples are glucose-rich, indicating the nature of these polysaccharides to be glucan-based, with other neutral sugars. The RPS of *Synechocystis* sp. is composed of four main monosaccharides Glucose (Glc), Galactose (Gal), Rhamnose (Rha) and Mannose (Man), while traces (molar % < 1%) of Xylose (Xyl), Fucose (Fuc) and Ribose (Rib) were detected. The RPS of the Chroococcales cyanobacterium LEGE 19970 presents in the same way four main monosaccharides Glc, Rha, Gal and Man with traces of Xyl and Fuc.

**Table 2:** Monosaccharides composition of cyanobacterial RPS.

Strain	Monosaccharides composition (Molar %)						
	Rha	Gal	Glc	Xyl	Fuc	Man	Rib
*Synechocystis* sp. LEGE 07367	9.3	13	67.3	Traces	Traces	8.4	Traces
Chroococcales cyanobacterium LEGE 19970	30	12	50	Traces	Traces	4	

Monosaccharides composition is expressed in Molar % (*n* = *3*) Traces were considered when molar % lower than 1%.

### Molecular weight (Mw) distribution

The Mw distribution of the RPSs was determined by HPLC-SEC/MALS analysis, experiments were carried out in 0.1 mol L^−1^ NaNO_3_ (SM Figure S3). The polysaccharides were eluted between 16 and 23 mL which are typical range for high molecular weight fractions and exhibited distinct elution profiles. Effectively, the LS signal of *Synechocystis* sp. LEGE 07367 does not return to the initial level at the end of the elution, which indicates a tailing phenomenon of the compound analyzed, unlike the RPS of Chroococcales cyanobacterium LEGE 19970. Thus, this indicates non-ideal elution, characterized by slow elution of molecules regardless of their hydrodynamic volume which can affect the separation of entire molecular weight in the samples. In order to improve the dissolution of RPS, different parameters were tested (concentration, temperature, high homogenization).

The concentration detector (refractive index detector) and the dn dc^−1^ used (0.150) allowed to calculate the samples recoveries compared to the original mass of the injected sample. Sample recoveries obtained from *Synechocystis* sp. LEGE 07367 was lower (< 10%) than Chroococcales cyanobacterium LEGE 19970 (around 30%). Filtration of the sample through a small pore size filter (≤ 0.45 μm) may result in sample loss in the case that high molecular weight RPS may be present as aggregates. These low recoveries might also be due to material loss caused by sample adsorption due to interaction with the column packing material, as suggested by the LS signal.

A summary of the Mw distribution of *Synechocystis* sp. LEGE 07367 and Chroococcales cyanobacterium LEGE 19,970 RPS is shown in Table 3. Both RPS presented a polydispersity index of the same magnitude. The average Mw of the RPS of *Synechocystis* sp. LEGE 07367 is higher than Chroococcales cyanobacterium LEGE 19970 RPS. A subtle UV signal was detected in the RPS fractions of both cyanobacteria (data not shown).

**Table 3:** Average Mw, polydispersity index (D) and Mw distribution of cyanobacterial EPS.

Strain	Mw (g mol^−1^)	Mn	Đ
*Synechocystis* sp. LEGE 07367	2.91 × 10^6^	2.5 × 10^6^	1.96
Chroococcales cyanobacterium LEGE 19970	6.36 × 10^5^	5.6 × 10^5^	1.14

## Cyanotoxin detection

Cyanobacterial toxins were evaluated for both RPS-producing strains (Table 4). The polymerase chain reaction (PCR) detection was carried out for six genes associated with the biosynthesis of four cyanotoxins (Microcystin, Saxitoxin, Cylindrospermopsin and Anatoxin). Although no genes were detected for both freshwater strains, the stx gene was initially detected for *Synechocystis* sp. LEGE 07367. Sequencing of the amplified PCR product did not correspond to the saxitoxin-related gene. To conclude the absence of this toxin, an ESI–LC–MS/MS analysis was carried out for both biomass and supernatant of *Synechocystis* sp. LEGE 07367 culture. STX standard solution showed a retention time (RT) of 3.25 min and mass fragments of 282, 266, 240, 221, 204 and 144 m/z (Figure S4). The *Synechocystis* sp. LEGE 07367 cell extracts and media total ion chromatograms were analyzed in the LC signal region with RT 2–10 min and all the collison induced dissociation (CID) mass spectrum did not show the fragment pattern expected for STX or Neosaxitoxin (Figure S4 C–F). Together, the evidence supports the absence of STX and Neosaxitoxin in the cells and media of *Synechocystis* sp. LEGE 07367. Therefore, both freshwater strains are free of known cyanotoxins.

**Table 4:** Detection of genes involved in microcystin (MC), saxitoxin (STX), cylindrospermosin (CYL)and anatoxin (ANA) via PCR and ESI–LC–MS/MS for saxitoxin.

LEGE-CC strain code	PCR				ESI–LC–MS/MS
	MC	STX	CYL	ANA	STX
*Synechocystis* sp. LEGE 07367	–	A	–	–	–
Chroococcales cyanobacterium LEGE 19970	–	–	–	–	Nd

*Nd* non-determined, *A* false positive.

## Discussion

This study has explored a novel way in bioprospecting EPS producing strains by subjecting them to industrially relevant conditions (diel light and temperature).

A screening study comprising rich biodiversity was carried out in the Roscoff Culture Collection, with five different orders explored, broadening the knowledge of cyanobacterial RPS producers under constant light and temperature. *Synechococcus* sp. RCC 2380 was highlighted as a RPS-producing strain showing a fair productivity of 4 mg L day^−1^^27^. Our study contributed to increase the screened taxonomic diversity over five orders from several genera. When bioprospecting EPS producing strains reports have consistently focused on EPS as single product^28–33^ while some included biomass production^34–36^ and few biochemical parameters^37^. Using diel light and temperature to simulate real climate is crucial to address the industrial implementation of these photoautotrophic factories^38^. Understanding which strains adapts better to these conditions can help to reduce energy input for process control. For that, biomass productivity is a valuable metric to assess the potential of these strains. *Spirulina* sp. LEB-18 RPS-producing when grown in a 250L raceway tank showed peak biomass productivity of 0.02 g L^−1^day^−1^^39^. Our study showed maximal biomass productivities around 0.2 g L^−1^ day^−1^ however, these are expected to lower when volume of cultivation increases. Harnessing other valuable metabolites beyond EPS can provide multi-product from one single cultivation and valorise the overall bioprocess. Some studies have explored EPS and pigments^40,41^ or the production of bioethanol^42^. Our study has focused on the macromolecular composition of the biomass which provides a general overview of each cyanobacteria biochemical profile. *Cyanobium* sp. LEGE 06133 has been highlighted as a rich source of pigments (phycobiliproteins and carotenoids) with a biomass productivity of 0.14 g L^−1^ day^−1^ under optimized conditions^43^. The two most productive *Cyanobium* strains, showed higher biomass productivity than the one grown in optimized conditions. The other most productive strains screened in this work belong to the *Synechocystis* genus. *Synechocystis salina* has been reported as a valuable source of antioxidant molecules^44^ and polyhydroxyalkanoates^45^, making them potential candidates for a biorefinery concept^46^.

The demand for alternative protein sources is rising, and cyanobacteria are in the spotlight as one of them^47^. The present study evaluated the protein content of the following genus for the first time: Chroococcales cyanobacterium LEGE 17607, *Altericista* sp. LEGE 17690, Synechococcales cyanobacterium LEGE 19969, Chroococcales cyanobacterium LEGE 19970, *Tolypothrix* sp. LEGE XX280 and Nostocales cyanobacterium LEGE 18510. Our studied strains had relatively lower protein content in comparison to other well-studied cyanobacterial genera, namely *Anabaena* (43–56% DW), *Arthrospira* (17–72% DW), *Aphanizomenon* (62% DW), *Synechococcus* (46–63% DW)^48^. Although there are different routine methodologies for protein quantification (nitrogen analysis, colorimetric assays, and sum of anhydroamino acids), inconsistencies have been shown between the results obtained with one or the other, so conclusions about the actual protein content must be careful^48^.

The model cyanobacteria *Synechocystis* sp. PCC 6803 has a reported protein content varying from 32 to 57% DW^49^, similar to that found for *Synechocystis* strains (34–40% DW). The *Cyanobium* sp. LEGE 06113 was characterized by low protein content^50^, following the one here obtained for the *Cyanobium* strains.

The most productive strains showed similar lipid content. Nordic freshwater cyanobacteria grown under synthetic wastewater showed similar total lipid content (6.7 and 23.5% DW)^51^. The freshwater *Cyanobium* sp. CACIAM06 showed 5.5% DW of lipid content^52^, lower than the one observed for the freshwater *Cyanobium* sp. LEGE 15611.

The different ash content obtained for marine strains compared to freshwater strains is related to the higher concentration of salts in the marine medium.

Carbohydrate accumulation is thought to work as a carbon sink from photosynthesis whenever nutrient-depleted conditions occur. In addition, under non-depleted nutrient conditions, cyanobacteria can be composed of low carbohydrate content (10–30% DW)^53^. Our results showed 5 out of 7 cyanobacteria with carbohydrate content higher than 30% DW, whereas the other two have levels near this virtual maximum, indicating that the cultures might be at the end of the growth phase in nutrient-depleted conditions. In the present work, a higher average % DW of carbohydrates was found for freshwater strains in comparison with marine strains. However, there is not enough bibliography to discuss this aspect.

The NMDS stress value was considerably low, indicating a very good fit. This is probably due to the relatively low number of objects in the analysis and dimensions to establish the dissimilarities. As a result, the reduction to a 2-dimensional dissimilarity plot is very simple and robust.

The PCA analysis showed a negative correlation between carbohydrate and protein content. A previous study on cyanobacterial biomass have found similar results^54^. The negative correlation between carbohydrate and lipid content was described for seven cyanobacteria strains, which agrees with previously obtained results^55,56^. To date, no study has explored the correlation between lipid and protein content in cyanobacteria, whereas our study indicates this positive correlation for both strains of marine and freshwater environments. Our NMDS are in accordance with the PCA correlation observed.

The carbohydrate levels obtained in this study correspond to intracellular carbohydrates and CPS since biomass harvest did not undergo any washing step. Thus, we can assume that *Cyanobium* sp. strains (LEGE 06140 and LEGE 15611) are either rich in intracellular carbohydrates or CPS. Depending on their location, these carbohydrates could be feedstock for bioenergy purposes^57^ or, in the case of CPS, the production of extracts for cosmetics or cosmeceuticals^58^.

The EPS detection method employed in this work has been frequently applied to detect cyanobacterial EPS^59–61^. As observed in the microphotographs there is not a clear distinction between the RPS-producing strains and the non producing strains, since this stain is not molecular weight specific lower sizer fractions can be also stained however, the methodology applied in this work targets high molecular weight. Therefore, the staining approach among the most productive strains served as an indicator of the EPS architecture involving the cyanobacterial cells. Cyanobacteria rich metabolism allows the use of different nutrient sources which affect their physiology and performance^56^. The use of extract sea mud in a f/2 modified media promoted an EPS productivity of 1.3 g L^−1^ day^−1^ on *Cyanothece* genus strain however, no insights into biomass productivity or composition was mentioned^30^. Despite the surprising EPS productivity the impact on the use of this resource at large scale must be considered.

Some of the most studied cyanobacteria for EPS production were grown under diazotrophic conditions^28,31,33,36^. Diazotrophic conditions promoted higher RPS productivities than the ones obtained in this study namely for axenic cultures of *Nostoc* sp. PCC 7413 and *Nostoc* sp. PCC 8109 around 47 mg L^−1^ day^−1^ and 30 mg L^−1^ day^−1^, respectively^28^. The absence of nitrogen source can have a huge economical impact on nutrients associated costs however, the sensitivity of nitrogenase enzyme to oxygen is a factor affecting the growth rate of cyanobacterial biomass and must be addressed at a strain specific level before considering industrial exploitation^62^. Our study screened non nitrogen-fixing cyanobacteria from a broad range of cyanobacterial orders using an industrial optimized version of f/2 media. This formulation is expected to be cost competitive in comparison to more complete versions of cyanobacterial growth media. The RPS productivities under Portuguese climate were considerably higher when compared to those obtained from representants of Nostocales, Chroococcales, Synechococcales, Oscillatoriales, Spirulinares Pleurocapsales orders which were mostly grown under stable light and temperature conditions^27,29,32,34,35,37,63^. Both obtained RPS are glucan-based, in accordance with EPS composition described for cyanobacteria^64^. However, no uronic acid was detected, which represents a less common trait found in these EPS^9,65^. Two common uronic acids (GlcA and GalA) were used as standards with a detection limit of 0.5 µg µL^−1^, which should be enough to detect their presence. Less common uronic acids could be expected from cyanobacteria, so further experiments are required to confirm the absence of these sugar acids. Performing a reducing step prior to derivatization with TMS, NMR 13C, or ionic chromatography without derivatization are some of the techniques to be applied.

Chroococcales cyanobacterium LEGE 19970 showed four main monosaccharides, among which 80% were glucose and rhamnose, suggesting the presence of a Rhamnoglucan RPS. These two monosaccharides have been described to be ubiquitous in cyanobacterial EPS^64^.

*Synechocystis* sp. LEGE 07367 monosaccharides composition has shown a distinct composition when compared to *Synechocystis* PCC 6803 and PCC 6714 despite glucose was a major monosaccharide, a total of 11–12 monosaccharides and uronic acid were identified^66^. PCC 6714 RPS had a glucose content more similar to *Synechocystis* sp. LEGE 07367 RPS. Additionally, the wild type of PCC 6803 was shown to have more balanced amounts of hexoses, while derived mutants have shown increasing amounts in hexoses, particularly of glucose which seems to be closer to the RPS composition of LEGE 07367^67,68^. Genetic differences between these strains or the culture conditions applied could be responsible for the observed variability in monosaccharides composition.

Average molecular weight found for both RPS are within the ranges described for cyanobacterial EPS^69^. *Synechocystis* sp. LEGE 07367 RPS average molecular weight is in agreement with the molecular weight proposed for *Synechocystis* sp. PCC 6803^70^.

A smaller RPS fraction was found for the Chroococcales cyanobacterium LEGE 19970 averaging 636 kDa. The absence of a purification step for *Synechocystis* sp. LEGE 07367 RPS could influence the polydispersity index. The conductivity and salinity parameters of the RPS were suitable for monosaccharides analysis and thus, a broader estimated range of molecular weight was obtained. Mota and collaborators^18^ have observed that the RPS from *Cyanothece* CYY010 has a diverse relative abundance whereas most polymeric fractions ranged from 50 kDa to 343.2 Da, yet 30% of the RPS fraction contained MDa size polymers. The diversity on monosaccharides and number of molecular weight fractions clearly indicate the complexity of the RPS. In addition, these structural features found can be affected by the extraction methodologies and the analytical methods applied^9,71^. Purification steps can help to hinder the structures of these polysaccharides and better understand how related to each other they are.

The proteic moieties detected in both RPS fractions is commonly described in cyanobacterial RPS^9,72^.

Cyanotoxins are responsible for negatively impacting the ecosystems, however, they could also be a high added value product for standards market or biomedical applications^73^. Some *Synechocystis* strains were found to be potent neurotoxin and hepatotoxin producers^74^. However, in strain *Synechocystis* sp. LEGE 07367 the absence of cyanotoxins was confirmed, as well as in strain Chroococcales cyanobacterium LEGE 19970. This positions their interest for certain biotechnological applications. Certainly, process-oriented screenings of unexplored cyanobacterial strains will play a role in the implementation of cyanobacteria-based bioprocess for the biotechnology sector in Portugal.

## Conclusions

The screening of cyanobacteria using diel light and temperature of Portuguese climate allowed to distinguish strains from a rich taxonomic group according to their performance, biomass composition, ability to excrete polysaccharides and produce cyanotoxins.

Two cyanotoxin-free cyanobacterial strains, *Synechocystis* sp. LEGE 07367 and Chroococcales cyanobacterium LEGE 19970, were identified as fast growing (0.14 and 0.12 g L^−1^ day^−1^, respectively) sourcing soluble glucan-rich RPS of high molecular weight 2910 kDa and 636 kDa, respectively. Their biomass composition rich carbohydrates (> 42% DW) and in protein (> 36% DW) hold potential for a wide range of applications.

Fast-growing yet non-RPS-producing strains [*Synechocystis salina* (3) and *Cyanobium* strains (2)] should be considered for the biorefinery concept due to their rich carbohydrate and/or protein content, which together accounted for 65–83% DW.

This work provides an entry point for future outdoor cultivation of cyanobacteria with natural sunlight and reduced temperature control. The LEGE-CC showed its potential as a source of unexplored diversity, after the detection of potentially new genera of cyanobacteria, including one RPS producer.

## Materials and methods

### Strains pre-selection and cultures maintenance

Sixty-three cyanobacterial strains were provided by LEGE-CC. At this point strains were kept under culture collection conditions and evaluated through light microscopy with Alcian Blue staining for neutral polysaccharides^34^. Briefly, strains (stationary phase) were kept under a 12/12 h Light/Dark cycle (L/D), light intensity < 20 µmol m^−2^ s^−1^ (fluorescent light, Acardia),19 °C, and maintained in a strain-dependent culture media: Z8 medium^75^, Z8 medium supplemented with 25‰ synthetic sea salts (salt Tropical Marin, Berlin, Germany) and 1‰ with vitamin B12^75^ or BG11_0_^76^ Cultures and Alcian blue formulations were mixed in a 1:1 ratio, and using a Leica DMLB light microscope coupled to a Leica ICC50 HD digital camera (Leica Microsystems, Germany), the presence of neutral polysaccharides was observed.

Then, the twenty-five strains were adapted to Allmicroalgae’s industrial medium, based on Guillard’s F/2 medium^77^, containing 10 mM of nitrogen and 12.5 mM of iron. Cultures were kept at 25 °C, 18/6 h L/D cycle, 10–30 µmol m^−2^ s^−1^. Marine strains were grown under the same medium but supplemented with magnesium water and NaCl to reach salinity of 30 g L^−1^.

### DNA extraction, amplification (PCR) and sequencing

Genomic DNA was extracted using the Genomic DNA Mini Kit (Invitrogen, Waltham, MA, USA), according to the manufacturer's instructions for Gram-negative bacteria. The 16S rRNA gene sequence (specific primer pairs available in SM Table 3) was obtained upon PCR amplification following the same conditions^78^. The sequencing was performed at GATC Biotech (Ebersberg, Germany), and the nucleotide sequences obtained were manually inspected for quality and assembled using the Geneious Prime 2021.2.2 software (Biomatters Ltd., Auckland, New Zealand). Sequences were checked for possible chimera formation using the DECIPHER software^79^ before any phylogenetic analysis. Sequences obtained were inserted in the BLASTn (Basic Local Alignment and Search Tool for Nucleotides) database, and the results were analyzed. The sequences associated with this study were deposited in the GenBank database under the accession numbers OR046505 to OR046517.

### Phylogenetic analysis

A total of 183 sequences were used in the final analysis, including 2 strains of *Gloeobacter violaceus* as outgroup, 159 sequences of cyanobacteria including type and reference strains retrieved from GenBank (National Center for Biotechnology Information, NCBI, Bethesda, MD, USA), and 22 sequences of LEGE-CC strains^23^. Multiple sequence alignment was constructed using MAFFT v7.450^80,81^ and sequences were manually proofread and edited. The best substitution model for ML-based analyses was chosen using jModelTest 2 software^82^ using the Akaike information criterion. Maximum likelihood (ML) analysis was carried out using the substitution model GTR + G + I with 1000 bootstrap resampling replicates using the IQ-TREE 2 software^83^. The final phylogenetic tree was edited on iTOL (Interactive Tree of Life)^84^ and Inkscape 1.2^85^.

### Experimental set-up and photobioreactor operation

Prior to cultivation in PBR, strains were placed in an orbital shaker SHK-2013 (100 rpm) for 7 days ALGAETRON AG230 (PSI Instruments) illuminated with cool white LED at 70 µmol m^−2^ s^−1^ and 22 °C under 18:6 L/D cycle. PBR cultivation was carried out in ALGEM systems (Algenuity, UK) with 25 (v/v %) inoculum, comprising 0.2 L cultivation volume. ALGEM features a geographic environment modelling that can be used to precisely simulate a specific environment. The location of Allmicroalgae S.A. production plant (39.652936 N, − 8.988986 W), was used as virtual site of experimentation, while diel light and temperature for the month of May simulated spring. The pH was set to 8 and controlled on demand through the injection of a 30% CO_2_–air mixture. Agitation was set constant at 100 rpm, and no aeration was provided. Cultivation was carried out for 15 days, and samples were taken every 2 or 3 days for culture monitoring.

### Biomass harvest and supernatant processing

Biomass harvest was carried by centrifugation (Herareus Megafuge 16R, Germany) at 15000 g for 10 min at 4 °C and stored for further analysis. The supernatant was then subjected to a second centrifugation step at 18,000 g for 20 min at 4 °C, to remove persistent biomass and remaining cell debris. Thereafter, the supernatant was precipitated in cold ethanol at 3:1 (v/v %) and kept overnight under − 20 °C. A mesh and a strainer were used to recover the precipitate, which was then dried and stored.

### Cyanobacterial growth performance

Biomass productivity was calculated from biomass concentration obtained on days two and fifteen. Briefly, the biomass concentration was determined by dry cell measurements carried under mild vacuum filtration. Ammonium bicarbonate (0.5 M) was used to wash the GF/F glass microfibre filters (Whatman). Filters (F) were previously dehydrated at 60 °C for at least 24 h proceeded by silica chambers for 1 h, before (F1) and after (F2) biomass retention. Dry cell weight corresponding to biomass concentration was obtained in g L^−1^ and calculated according to Eq. 1. Biomass productivity was calculated according to Eq. 2.

1 $$Biomass\;concentration\;C_{x} \left( {\frac{{\text{g}}}{{\text{L}}}} \right) = \frac{F2 - F1}{{Volume\;of\;culture}}$$

2 $$Biomass\;productivity\;R_{x} \left( {\frac{g}{L \cdot day}} \right) = \frac{{\Delta C_{x} }}{days\;of\;cultivation}.$$

### Biomass proximate composition

Protein content was estimated for the fifteen strains that grew under the simulated environment by CHN elemental analysis, according to the procedure provided by the manufacturer, using a Vario el III (Vario EL, Elemental Analyzer system, GmbH, Hanau, Germany). The final protein content was determined by multiplying the percentage of nitrogen by 5.22^86^.

Lipid, ash and carbohydrate contents were only estimated for the most productive strains. The lipid content was estimated using the Bligh & Dyer method^87^ described in with minor modifications^88^. Briefly, freeze dried cyanobacterial samples (7–35 mg) were mixed with ~ 0.6 g of glass beads and extracted with methanol through bead-milling using a Retsch MM 400 mixer mill at 30 Hz for 3 min. The tubes were centrifuged (10,000 g), and the supernatants were collected to new vials. The pellets suffered a second extraction, and both methanol supernatants were pooled. Chloroform and water were added to the methanol (2:1:2), and the samples were vortexed for 5 min. Afterwards, the samples were centrifuged (2500 g for 10 min) to obtain a biphasic system, and the lipid extract (chloroform) was separated. A known volume of the extracts was transferred to pre-weighed tubes, evaporated and weighted in order to determine the lipids gravimetrically. The ash content was determined by burning the freeze-dried biomass (approximately 35 mg) in a furnace (J. P. Selecta, Sel horn R9-L, Barcelona, Spain) at 550 °C for 6 h^89^. The carbohydrate content was determined by difference of the remaining macronutrients.

### RPS purification

Raw RPS samples were purified when necessary, using ultrafiltration membranes (cut-off 50 kDa) by using AMICON Ultra-4 Centrifugal Filter Unit. Briefly, raw RPS samples were solubilized in ultra-pure water and centrifuged for 10 min at 5000 rpm and room temperature. Conductivity and salinity were followed (HANNA Instruments) to track the presence of salts. When a 0.2% (w/v) RPS solution exhibited low conductivity (< 500 µS cm^−2^), it was considered appropriate for further analysis.

### RPS partial characterization

#### Monosaccharides composition

Gas chromatography-mass spectrometry (GC–MS) by Eletrocnic Impact (EI) was used to determine the monosaccharides composition. Briefly, 1 mL of 2 M trifluoroacetic acid (TFA) was used for the dissolution of 10 mg of RPS. The hydrolysis step was performed with a thermoblock at 120 °C for 90 min followed by solvent evaporation under Nitrogen atmosphere at 60 °C. Trimethylsilylation derivatization method was performed as proposed by.Pierre and collaborators^90,91^. L-Rhamnose (L-Rha), L-Fucose (L-Fuc), L-Arabinose (L-Ara), D-Xylose (D-Xyl), D-Mannose (L-Man), D-Galactose (D-Gal), D-Glucose (D-Glc), D-Glucuronic acid (D-GlcA), D-Galacturonic acid (D-GalA), D-Glucosamine (D-GlcN), and D-Galactosamine (D-GalN) were used as standards. The samples were injected on a Shimadzu Nexus Gas Chromatrography 2030 system coupled to a GCMS-QP2020NX (Shimadzu, Japan), equipped with an OPTIMA-1MS Accent column (Macherey–Nagel, 30 m, 0.25 mm, 0.25 µm. Hydrogen flow rate was set at 1.75 mL min^−1^. (7.8 psi). The first temperature ramp was set to 100 °C during 2.75 min then an increase until 200 °C was achieved at 8.4 °C min^−1^. The second temperature ramp was raised until 215 °C during 0.95 min at a constant pace. The electronic ionization (EI, 70 eV) was carried at 150 °C, and the target ion was fixed at 40–800 m/z. The solvent cut time was set at 5 min, the split ratio was 50:1, and the temperature of the injector was 250 °C. The relative molar proportions were determined according to the area normalization. MestReNova Software 7.1.0–9185 (Mes-trelab Research, Coruña, Spain) was used to analyze the data.

#### Mw estimation

High pressure size exclusion chromatography (HPSEC) coupled with three detectors: Multi-angle laser light scattering detector (MALS, Mini-DAWN TREOS II, Wyatt Technology Corp., Santa Barbara, CA, USA), differential refractive index (DRI) detector (RID-10 A, Shimadzu, Duisburg, Germany) and UV–vis detector (SPD-20A, Shimadzu, Duisburg, Germany) were used to determine molecular weight of the samples. The HPSEC line consisted of an SB-G guard column and three columns in series (SB-806 HQ, SB-804 HQ and SB-803 HQ). The system was eluted with NaNO_3_ 0.1 M and NaN_3_ 2.5 mM, filtered through a 0.02 µm, 47 mm membrane filter (Anotop 47, Whatman, Maidstone, England), and carefully degassed. RPS samples were previously prepared by stirring in the elution buffer for at least 24 h at 60 °C and homogenized in Ultraturrax at 19,000 rpm for 20 s. Lastly, samples were filtered through a 0.45 μm syringe filter (Grace Altech, Maryland, USA). Injection was carried through a 100 µL full loop and the elution was performed with a flow rate of 0.5 mL min^−1^. Data were evaluated using ASTRA 7.2.2 software.

### Cyanotoxin detection

#### Molecular methods

The cyanotoxin detection was carried by molecular methods. Genomic DNA was obtained as in “DNA extraction, amplification (PCR) and sequencing” section. Genes mcyE, mcyA, were targeted for microcystin (MCs) and nodularin (NOD) production potential, sxtA, sxtG and sxtI for saxitoxin (STX), and cyrJ cylindrospermosin (CYN) and anaC-gen for anatoxin (ANA) using specific primer pairs available in SM Table 3.

#### Extraction, SPE and ESI-LC–MS/MS analysis

Both cells and media were analyzed by ESI–LC–MS/MS to chemically confirm the *Synechocystis* sp. LEGE 07,367 toxicity of the Saxitoxin positive molecular outcome. Cells (fresh pellets) were collected after centrifugation, separated from the media (lyophilized) and extracted with some modifications^92^. The cells were dissolved in 15 mL of a methanolic solution (MeOH:H_2_O, v/v 20:80), sonicated to lysis (ice, 95% amplitude, 5 min) and centrifuged (5000 g, 4 °C, 2 min). This extraction was repeated twice, and all the supernatants were pooled together prior complete rotavapor dryness of the methanol.

Both cell extracts and lyophilized media were resuspended in 200 mL of ultrapure water (LC–MS grade) before being submitted to solid phase extraction using Supelclean ENVI-carb cartridges (250 mg, 6 mL, SUPELCO, Sigma-Aldrich, USA).

ENVI-carb cartridge was wetted with 6 mL of dichloromethane (DCM), 6 mL of MeOH, and 6 mL of ultrapure water (LC–MS grade). Samples (200 mL) were loaded at ~ 10 mL min^−1^ via a 12-port vacuum manifold (Waters, USA). Cartridges were then blown dry under nitrogen, eluted with 10 mL 60:40 MeOH:DCM (0.5% formic acid) into 15 mL glass test tubes and dried under vacuum with a rotary evaporator. Final extract was then reconstituted with 500 µL 10:90 H_2_O:MeOH (0.1% formic acid) and transferred to a 1.5 mL vial for LC–MS/MS analysis.

Samples were injected in a Liquid Chromatograph Thermo Finnigan Surveyor HPLC System (Thermo Scientific, MA, USA), coupled with a Mass Spectrometry LCQ Fleet™ Ion Trap Mass Spectrometer (Thermo Scientific, MA, USA). The program used for data acquisition and processing is XcaliburTM version 2. Mass Spectrometer Tune Method parameters optimization was performed thought direct injection of a Saxitoxin standard (CRM-00-STX, Lot 18-001, 99% purity, from Cifga, ES) solution of 1 ppm in10:90 H2O:MeOH (0.1% formic acid).

Mass Spectrometer operated in electrospray positive polarity mode using Full scan (30–1500 m/z) and Collision-Induced Dissociation (CID) at 300 m/z and 316 m/z, corresponding to Saxitoxin and Neosaxitoxin molecules ion precursors.

The spray voltage maintained at 7.0 kV; Capillary temperature at 350 °C; Capillary voltage and tube lens maintained at 22 and 50 kV, respectively. Nitrogen was used as sheath and auxiliary gas and collision energy at 35 eV.

Separation was achieved on a ACE Excel C18 (50 × 2.1 mm I.D., 1.7 μm, Batch: v19-3430, AVANTOR ACE ®, VWR, PT) kept at 30 °C, with a flow rate of 0.25 mL min^−1^. and injected volume was 10 μL in loop partial mode. The eluents used were methanol (A) and water (B) both acidified with formic acid at 0.1% (v/v). The gradient program started at 30% A, increasing to 60% A in 10 min, turning back to initial conditions in 5 min, equilibrating more 10 min with 30% A.

Under these conditions, the SAX retention time (RT) was 3.75 min and the limit of detection (LOD) and limit of quantification (LOQ) were 3 μg L^−1^ and 5 μg L^−1^, respectively. The precursor ion (m/z 300) and SAX reference fragment ions with m/z values of 282, 266, 240, 221, 204 and 144 were searched in the CID mode, to validate the presence or absence of the toxin.

### Statistical analysis

All the analyses were performed with R software^93^. The Kruskal–Wallis non-parametric test was performed with the *kruskal.test* function from the *stats* package, and the post-hoc *Nemenyi* test, with the function *kwAllPairsNemenyiTest* from the *PMCMRplus* package. Multi-variate analysis of variance (MANOVA) was performed with *manova* function from *stats* package, and multi-variate Shapiro–Wilks test with the function *mvShapiro.Test*, from package *mvShapirotTest*. Spearman correlation coefficient was calculated with *cor* function from *stats* package. Non-metric multidimensional scaling, employing Bray–Curtis distance, was performed with *metaMDS* function, from *vegan* package.PCA was performed with *PCA* function from *FactoMineR* package.

### Supplementary Information

Supplementary Information.

## Data Availability

Accession number of the genetic sequences were deposited in GENBANK and are available in supplementary material—Table S2. The datasets generated during and/or analysed during the current study are available from the corresponding author on reasonable request.
