# Supplementary material for: Bioprospecting for industrially relevant exopolysaccharide-producing cyanobacteria under Portuguese simulated climate
Source: Sci Rep. 2023 Aug 21;13:13561. doi: 10.1038/s41598-023-40542-6 (PMC10442320; doi:10.1038/s41598-023-40542-6)
Supplement: Supplementary file 1 — Supplementary Information. [file 41598_2023_40542_MOESM1_ESM.docx]

**Supplementary material**

**Bioprospecting exopolysaccharide-producing cyanobacteria under Portuguese simulated climate**

José Diogo Cruz^1,2^, Cédric Delattre^3,4^, Aldo Barreiro Felpeto^2^, Hugo Pereira^5^ Guillaume Pierre^3^, João Morais^1,2^, Emmanuel Petit^6^, Joana Silva^7^, Joana Azevedo^2^, Redouan Elboutachfaiti^6^, Inês B. Maia^8^, Pascal Dubessay^3^, Philippe Michaud^3^, Vitor Vasconcelos^1,2^

^1^ Faculty of Sciences, University of Porto, Rua do Campo Alegre, 4169-007 Porto, Portugal;

^2^ Interdisciplinary Center of Marine and Environmental Research (CIIMAR/CIMAR), University of Porto, Terminal de Cruzeiros do Porto de Leixões, Avenida General Norton de Matos, S/N, 4450-208 Matosinhos, Portugal

^3^ Université Clermont Auvergne, Clermont Auvergne INP, CNRS, Institut Pascal, F-63000, Clermont-Ferrand, France;

^4^ Institut Universitaire de France (IUF), 75005 Paris, France; cedric.delattre@uca.fr

^5^ GreenCoLab - Associação Oceano Verde, Universidade do Algarve, Campus de Gambelas, 8005-139, Portugal

^6^ UMRT INRAE 1158 BioEcoAgro, BIOlogie des Plantes et Innovation (BIOPI), Université de Picardie Jules Verne, IUT d’Amiens, Avenue des Facultés, Le Bailly, 80025 Amiens, France

^7^ Allmicroalgae Natural Products S.A, R&D Department, Rua 25 de Abril 19, 2445-287 Pataias, Portugal

^8^ CCMAR – Centre of Marine Sciences, University of Algarve, Gambelas, 8005-139 Faro, Portugal

^*^Corresponding author. Tel:

E-mail address:

**Table S1**- List of selected strains for Portuguese climate cultivation. Aspect of cultures under culture collection conditions and its respective qualitative detection of capsular polysaccharides (CPS) and released polysaccharides (RPS).

| **LEGE CODE** | **Aspect** | **Culture media** | **Alcian Blue Staining (Ethanol 50%)** | |
| --- | --- | --- | --- | --- |
|  |  |  | **CPS** | **RPS** |
| *Synechocystis salina* LEGE 00038 | homogenous, mucilaginous | Z8 + 25‰ Salt TM+ 1‰B12 | ++ | + |
| *Synechocystis salina* LEGE 00041 | smooth biofilm; mucilaginous | Z8 + 25‰ Salt TM+ 1‰B12 | - | + |
| *Synechocystis* sp. LEGE 07367 | homogeneous; mucilaginous | Z8 | ++ | +++ |
| *Synechocystis* sp. LEGE 06079 | homogeneous; mucilaginous | Z8 | ++ | + |
| Nostocales cyanobacterium LEGE 18510 | tuft forming | Z8 | + | + |
| *Tolypothrix* sp. LEGE 11397 | mucilaginous, | BG11_0_ | ++ | + |
| *Pegethrix* sp. LEGE 18685 | smooth biofilm, mucilaginous | Z8 | ++ | + |
| *Microcystis aeruginosa* LEGE 91353 | homogeneous; mucilaginous | Z8 | ++ | + |
| Chroococcales cyanobacterium LEGE 18573 | homogenous | Z8 | +++ | + |
| *Geminocystis* sp. LEGE 16574 | homogenous | Z8 | ++ | + |
| *Chalicogloea* sp. LEGE 18580 | homogenous | Z8 | ++ | + |
| Chroococcales cyanobacterium LEGE 19970 | homogenous | Z8 | ++ | + |
| Synechococcales cyanobacterium LEGE 19969 | homogenous | Z8 | ++ | + |
| Chroococcales cyanobacterium LEGE 17607 | homogenous | Z8 | +++ | + |
| *Cyanobium* sp. LEGE 15611 | homogenous | Z8 | + | ++ |
| No ID LEGE 17617 | homogenous | Z8 | ++ | + |
| D*esmonostoc muscorum* LEGE 12446 | homogeneous; mucilaginous | BG11_0_ | ++ | + |
| *Planktothrix* sp. LEGE XX280 | homogeneous; mucilaginous | BG11_0_ | ++ | + |
| *Cyanobium gracile* LEGE 09399 | smooth biofilm and aggregates | Z8 | ++ | + |
| *Cyanobium* sp. LEGE 06140 | homogeneous; mucilaginous | Z8 + 25‰ Salt TM+ 1‰B12 | ++ | + |
| Chroococcales cyanobacterium LEGE 16638 | Turf forming | Z8 | ++ | ++ |
| *Synechocystis* sp. LEGE 16643 | homogeneous; mucilaginous | Z8 | ++ | + |
| *Altericista* sp. LEGE 17690 | mucilaginous | Z8 | +++ | + |
| Chroococcidiopsidales cyanobacterium LEGE 14612 | Homogenous, mucilaginous | Z8 | + | + |
| *Synechocystis salina* LEGE 06099 | homogenous | Z8 + 25‰ Salt TM+ 1‰B12 | +++ | + |

Note: Subtitle for EPS detection: +++ abundant, ++ considerable, + present; - inexistent

**Table S2** – Identification list of selected strains for Portuguese climate cultivation. Strain placement at order level was done following (Komárek et al. 2014).

| **Strain identification** | **Order** | **Accession Number** | **Environment** | **Country** |
| --- | --- | --- | --- | --- |
| *Altericista* sp. LEGE 17690 | Synechococcales | OR046505 | Freshwater | Portugal |
| Chroococcales cyanobacterium LEGE 16638 | Chroococcales | OR046507 | Freshwater | Portugal |
| *Chalicogloea* sp. LEGE 18580 | Chroococcales | OR046506 | Freshwater | Portugal |
| Chroococcales cyanobacterium LEGE 17607 | Chroococcales | OR046508 | Freshwater | Portugal |
| Chroococcales cyanobacterium LEGE 18573 | Chroococcales | OR046509 | Freshwater | Portugal |
| Chroococcales cyanobacterium LEGE 19970 | Chroococcales | OR046510 | Freshwater | Portugal |
| Chroococcidiopsidales cyanobacterium LEGE 14612 | Chroococcidiopsidales | OR046511 | Freshwater | Portugal |
| *Cyanobium gracile* LEGE 09399 | Synechococcales | KU951680 | Freshwater | Portugal |
| *Cyanobium* sp. LEGE 06140 | Synechococcales | KU951697 | Marine | Portugal |
| *Cyanobium* sp. LEGE 15611 | Synechococcales | OR046512 | Freshwater | Portugal |
| D*esmonostoc muscorum* LEGE 12446 | Nostocales | KU951712 | Terrestrial | Portugal |
| *Geminocystis* sp. LEGE 16574 | Chroococcales | OR046513 | Freshwater | Portugal |
| *Microcystis aeruginosa* LEGE 91353 | Chroococcales | Not available | Freshwater | Portugal |
| No ID LEGE 17617 | Undefined | Not available | Freshwater | Portugal |
| *Pegethrix* sp. LEGE 18685 | Synechococcales | OR046515 | Freshwater | Portugal |
| *Planktothrix* sp. LEGE XX280 | Oscillatoriales | Not available | Freshwater | Portugal |
| Synechococcales cyanobacterium LEGE 19969 | Synechococcales | OR046516 | Freshwater | Portugal |
| *Synechocystis salina* LEGE 00038 | Chroococcales | KU951816 | Marine | Portugal |
| *Synechocystis salina* LEGE 00041 | Chroococcales | JADEVW000000000 | Marine | Portugal |
| *Synechocystis salina* LEGE 06099 | Chroococcales | JADEWK000000000 | Marine | Portugal |
| *Synechocystis* sp. LEGE 06079 | Chroococcales | HM217076 | Freshwater | Portugal |
| *Synechocystis* sp. LEGE 07367 | Chroococcales | KU951823 | Freshwater | Portugal |
| *Synechocystis* sp. LEGE 16643 | Chroococcales | OR046517 | Freshwater | Portugal |
| *Tolypothrix* sp. LEGE 11397 | Nostocales | KJ004418 | Freshwater | Portugal |
| Nostocales cyanobacterium LEGE 18510 | Nostocales | OR046514 | Freshwater | Portugal |


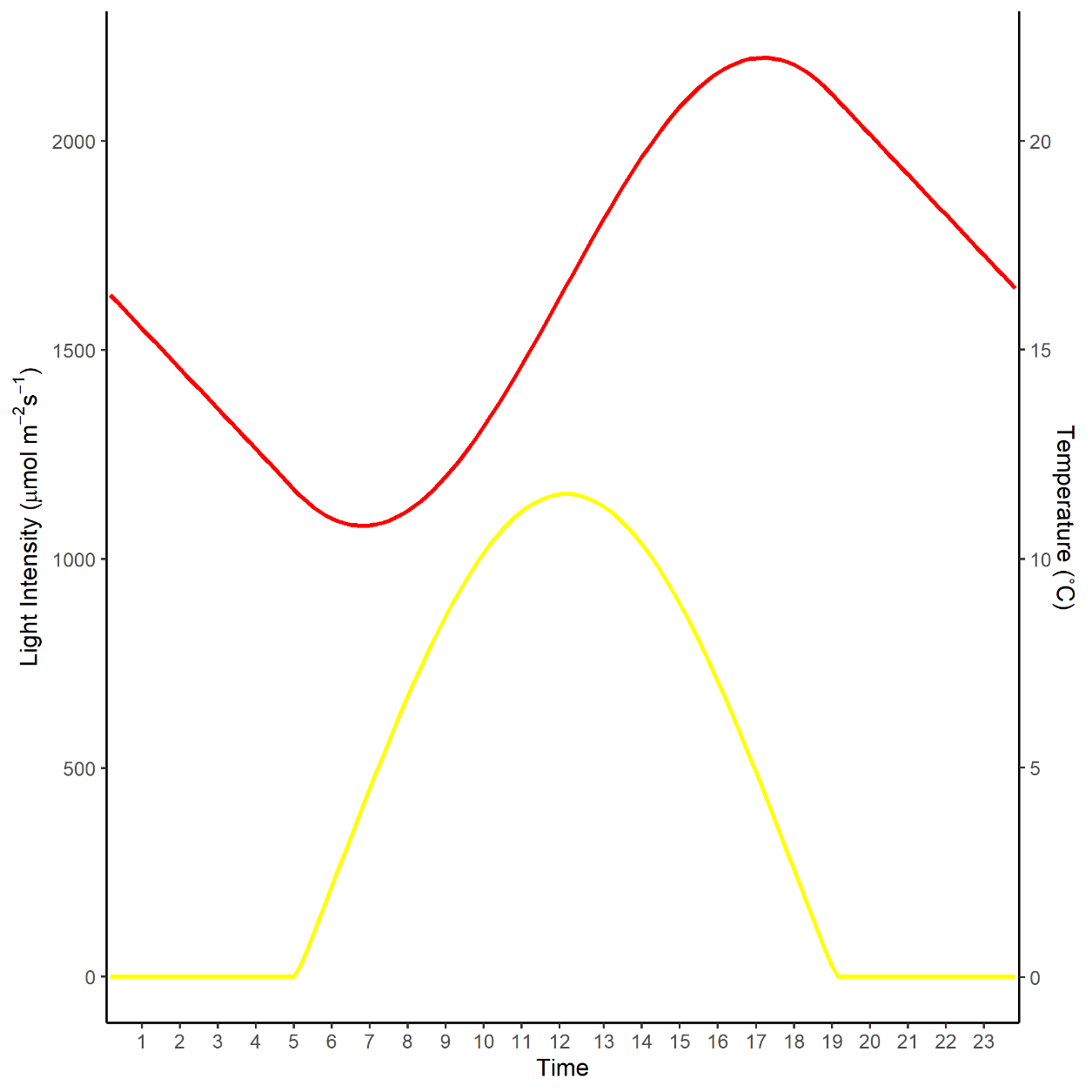


**Figure S1** – Diel light (-) and temperature (-) profile simulating spring (May) conditions of Allmicroalgae S.A. production plant (39.652936 N, −8.988986 W).

**Table S3** - Primers used to amplify and/ or sequence cyanotoxins biosynthesis genes.

| **Amplified gene** | **Primer** | **Fragment length (bp)** | **Sequence (5’-3’)** | **Literature** |
| --- | --- | --- | --- | --- |
| Microcystin/nodularin synthetase | HEPF | 472 | TTTGGGGTTAACTTTTTTGGGCATAGT | ^1^ |
|  | HEPR |  | AATTCTTGAGGCTGTAAATCGGGTT |  |
| Microcystin synthetase | mcyA-CDF | 297 | AAAATTAAAAGCCGTATCAA | ^2^ |
|  | mcyA-CDR |  | AAAAGTGTTTTATTAGCGGCTCA |  |
| stxA | sxtA855F | 648 | GCGTACATCCAAGCTGGACTCG  GACTCGGCTTGTTGCTTCCCC  AGGTCTTTGACTTGCATCCAA | ^3^ |
|  | sxtA1480R |  | GTAGTCCAGCTAAGGCACTTGC  GCCAAACTCGCAACAGGAGAAGG  AACCGGCGACATAGATGATA |  |
| stxI | sxtl682F | 200 | AGCGCTGCCGCTATGGTTGTCG | ^3^ |
|  | sxtl877R |  | ACGCAATTGAGGGCGACACCAC |  |
| cirJ | cynSulF | 584 | ACTTCTCTCCTTTCCCTATC | ^3^ |
|  | cylnamR |  | GAGTGAAAATGCGTAGAACTTG |  |
| anaC-gen | anaCgenF | 363 | TCTGGTATTCAGTCCCCTCTA | ^3^ |
|  | anaCgenR |  | CCCAATAGCCTGTCATCA |  |


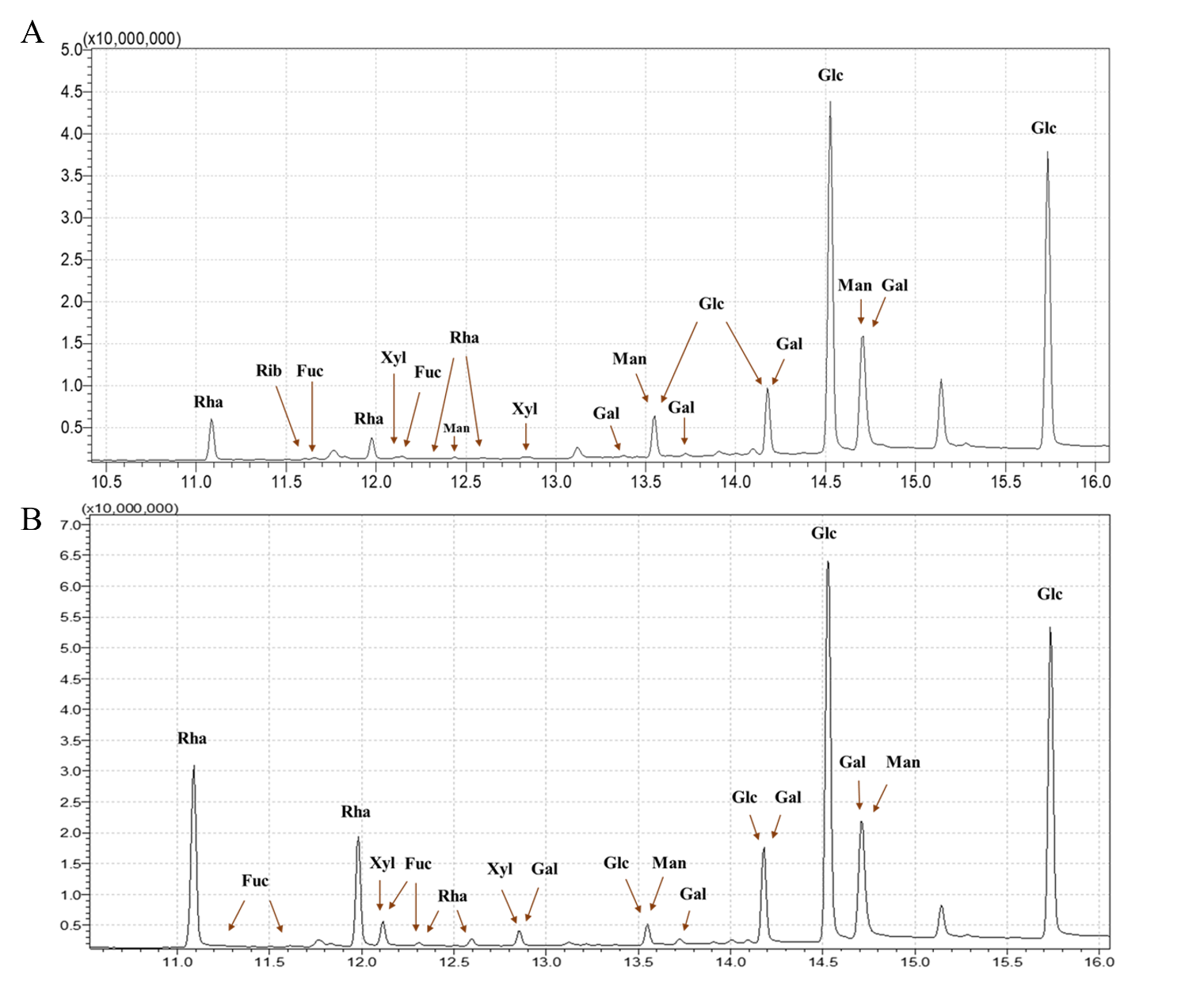


**Figure S2** – Total Ion Chromatogram (GC/MS-EI, 70 eV) of trimethylsilylated-*O*-glycosides from Chromatogram of hydrolysed RPS of A) *Synechocystis* sp. LEGE 07367 and B) Chroococcales cyanobacterium LEGE 19970.


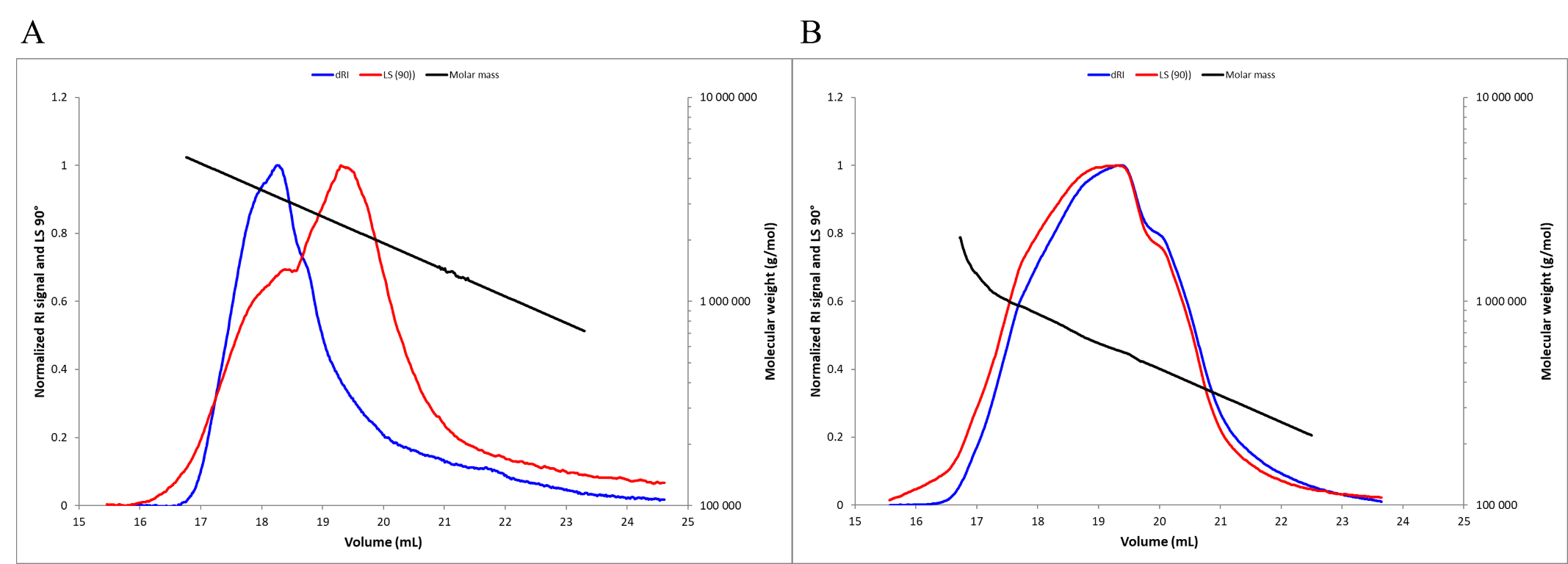


**Figure S3** - SEC-MALS chromatogram of A) *Synechocystis* sp. LEGE 07367 and B) Chroococcales cyanobacterium LEGE 19970 giving Mw (g/mol) versus V (ml) (black), RI signal (blue) and light scattering at 90° (red)...


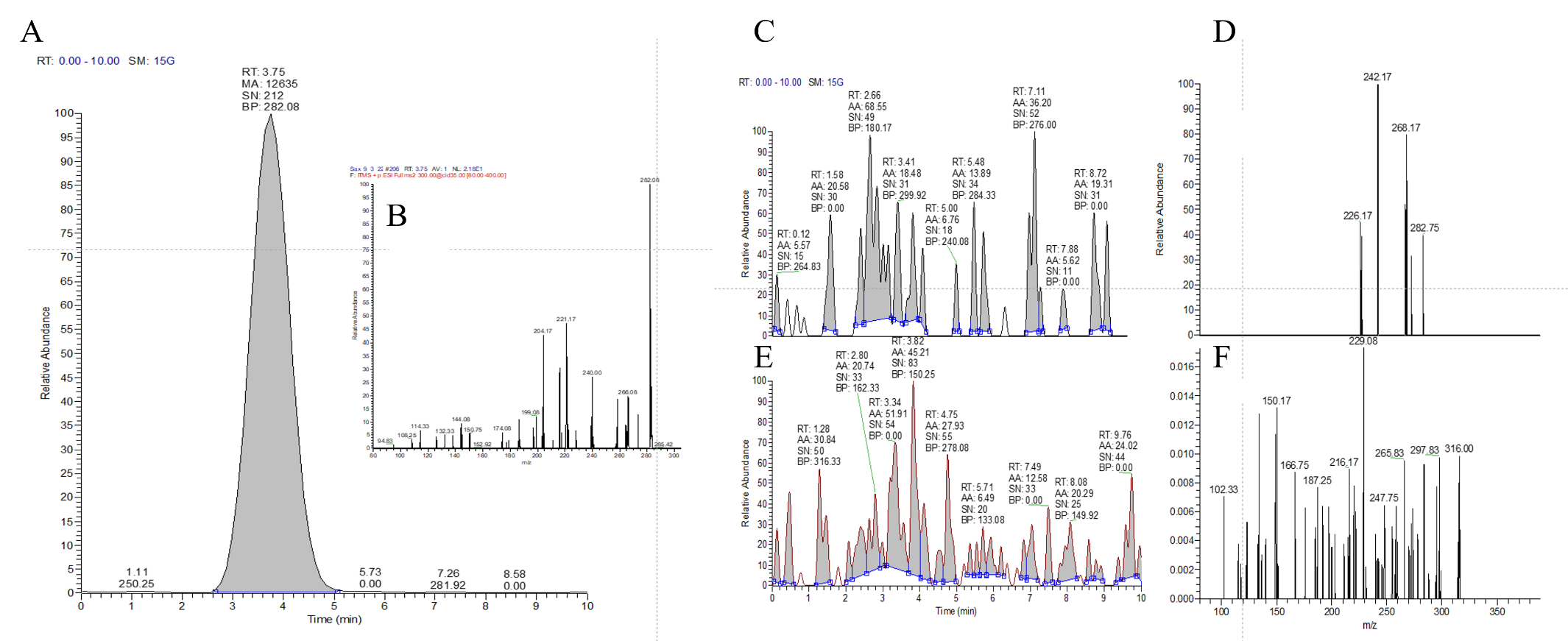


# **Figure S4** - Total ion chromatogram (A) of saxitoxin standard (100 ppb) with a retention time (RT) of 3.75 min and CID mass spectra (B) showing the characteristic saxitoxin molecule fragmentation in an Ion Trap Mass Spectrometer. Total ion chromatograms (C and E) of *Synechocystis* sp. LEGE 07367 solid phase extraction (SPE) treated cell extract and CID mass spectrum (D and F) showing no characteristic saxitoxin or Neosaxitoxin molecules fragmentation in an Ion Trap Mass Spectrometer.

**References**

1. Jungblut, A. *et al.* Diversity within cyanobacterial mat communities in variable salinity meltwater ponds of McMurdo Ice Shelf, Antarctica. *Environ Microbiol* **7**, 519–529 (2005).

2. Hisbergues, M., Christiansen, G., Rouhiainen, L., Sivonen, K. & Börner, T. PCR-based identification of microcystin-producing genotypes of different cyanobacterial genera. *Arch Microbiol* **180**, 402–410 (2003).

3. Kurmayer, R., Sivonen, K., Wilmotte, A. & Salmaso, N. Molecular tools for the detection and quantification of toxigenic cyanobacteria. (2017).
